# Enhancing the interpretation of real-world quality of life in patients with hormone receptor-positive/human epidermal growth factor receptor 2-negative advanced breast cancer enrolled in the POLARIS study

**DOI:** 10.1093/oncolo/oyaf281

**Published:** 2025-09-15

**Authors:** Gabrielle B Rocque, Joanne L Blum, Yan Ji, Timothy Pluard, John Migas, Shailendra Lakhanpal, Erin Jepsen, Eric Gauthier, Yao Wang, Monica Z Montelongo, Joseph C Cappelleri, Connie Chen, Meghan S Karuturi, Debu Tripathy

**Affiliations:** Hematology and Oncology, University of Alabama at Birmingham, Birmingham, AL 35233, United States; Baylor-Sammons Cancer Center, Texas Oncology, US Oncology, Dallas, TX 75246, United States; Oncology and Hematology, Health Partners Institute, St. Paul, MN 55101, United States; Breast Medical Oncology, Saint Luke’s Cancer Institute, Kansas City, MO 64111, United States; Mid-Illinois Hematology & Oncology Associates Ltd, Normal, IL 61761, United States; Hematology and Oncology, Saint Vincent’s Birmingham, Birmingham, AL 35205, United States; Medical Oncology, Novant Health, Winston-Salem, NC 27103, United States; Pfizer Inc., San Francisco, CA 94105, United States; Pfizer Inc., La Jolla, CA 92121, United States; ICON plc, Blue Bell, PA 19422, United States; Pfizer Inc., Groton, CT 06340, United States; Pfizer Inc., New York, NY 10001, United States; Department of Breast Medical Oncology, The University of Texas MD Anderson Cancer Center, Houston, TX 77030, United States; Department of Breast Medical Oncology, The University of Texas MD Anderson Cancer Center, Houston, TX 77030, United States

**Keywords:** advanced/metastatic breast cancer, HR+/HER2−, POLARIS, palbociclib, patient-reported outcomes, real-world evidence

## Abstract

**Importance:**

Patient-reported outcomes are recommended clinical outcome assessments of quality of life (QoL) by patient advocacy groups and regulatory agencies to gain a better understanding of treatment effectiveness, tolerability and safety.

**Objectives:**

In this study, we aimed to enhance and contextualize the interpretation of patient-reported scores on global health status (GHS) and QoL from the EORTC QLQ−C30 questionnaire into more meaningful terms using data from POLARIS.

**Design, Setting, and Participants:**

Patients ≥ 18 years of age who had a diagnosis of hormone receptor-positive/human epidermal growth factor receptor 2-negative (HR+/HER2−) advanced/metastatic breast cancer (ABC/mBC) were enrolled in the prospective, observational, multicenter, real-world POLARIS study.

**Intervention:**

Patients received palbociclib plus endocrine therapy as first-line, second-line or later line of therapy.

**Main Outcomes and Measures:**

Proportions of patients with “favorable” (numeric scores 5−7) and “unfavorable” (numeric scores ≤ 4) responses were determined at baseline and months 6, 12, and 18.

**Results:**

Between January 2017 and January 2023, 1250 patients were enrolled and received ≥ 1 palbociclib dose. EORTC QLQ−C30 GHS/QoL domain completion rates were 93.4%, 66.5%, 49.5%, and 42.3% at baseline and months 6, 12, and 18, respectively. For Question 29 (GHS), the proportion of patients with a favorable response significantly increased by ∼13% to 69.3% by month 6, which was maintained at month 12 (68.6%) and month 18 (70.0%). For Question 30 (QoL), the proportion of patients with a favorable response significantly increased by ∼9% to 74.5% by month 6, which was maintained at month 12 (75.0%) and month 18 (73.4%).

**Conclusions and Relevance:**

The proportions of patients with HR+/HER2− ABC/mBC indicating a favorable response on GHS and QoL questions of the EORTC QLQ−C30 increased early on after the start of palbociclib treatment and were preserved through month 18 across the overall study population and most evaluated subgroups. This simple interpretation of GHS and QoL scores is intended to enhance their meaning to benefit patients and other stakeholders.

**Clinical Trial Registration:**

NCT03280303; registered September 12, 2017.

Implications for Practice In this analysis of health-related QoL using the EORTC QLQ-C30 GHS/QoL domain, we have expressed the findings as prevalence of favorable and unfavorable responses. Our main study findings were that the proportions of patients with HR+/HER2− ABC/mBC indicating a favorable response on the GHS/QoL questions increased early on after the start of palbociclib treatment and were preserved through month 18 across the overall study population and most evaluated subgroups. The simple interpretation on GHS and QoL scores given here is intended to enhance their meaning for the benefit of patients and other stakeholders and inform care management.

## Introduction

While delaying disease progression and prolonging overall survival (OS) are very important to patients with advanced or metastatic breast cancer (ABC/mBC), it is increasingly recognized by both patients and healthcare providers that quality of life (QoL) while on treatment is also of significant importance.^1–3^ Patient-reported outcomes (PROs) are recommended clinical outcome assessments of QoL by patient advocacy groups and regulatory agencies to gain a better understanding of treatment effectiveness, tolerability, and safety.^1^^,^^4^^,^^5^ In the PALOMA-2 and PALOMA-3 trials, patient-reported QoL was an integral component of the benefit-risk assessment of palbociclib treatment in patients with hormone receptor-positive/human epidermal growth factor receptor 2-negative (HR+/HER2−) ABC/mBC.^6^^,^^7^ Among patients treated with palbociclib plus endocrine therapy (ET), QoL was maintained according to patient-reported assessment using the Functional Assessment of Cancer Therapy (FACT)–Breast and EuroQOL 5 dimensions (EQ–5D) in the PALOMA-2 trial and the European Organisation for Research and Treatment of Cancer Quality of Life Questionnaire Core 30 (EORTC QLQ-C30) in the PALOMA-3 trial.^6^^,^^7^

In the prospective, observational, multicenter, real-world POLARIS study (palbociclib in hormone receptor-positive advanced breast cancer: a prospective multicenter non-interventional study), a primary aim was to assess PROs among patients treated with palbociclib plus ET in routine clinical practice.^8^^,^^9^ The findings of POLARIS showed that patients with HR+/HER2− ABC/mBC who received palbociclib plus ET (*N* = 1250) maintained their QoL for at least 18 months while on treatment according to EORTC QLQ-C30 Global Health Status (GHS)/QoL scores, which averaged 64.0 at baseline, 69.3 at month 6, 70.1 at month 12, and 69.9 at month 18 while on treatment.^9^ These GHS/QoL findings from the POLARIS study align closely with those reported in the PALOMA-3 trial among patients treated with palbociclib plus fulvestrant (mean score baseline: 65.9; post-treatment: 66.1), in which the EORTC QLQ-C30 GHS/QoL score was also used to assess patient-reported QoL.^7^

The GHS/QoL summary score on the EORTC QLQ-C30 represents the average of the scores on Question 29 (Q29, GHS) and Question 30 (Q30, QoL) on which patients report their assessment using a 7-point Likert scale (1 indicating very poor GHS/QoL, up to 7 indicating excellent GHS/QoL); this raw score is then standardized by linear transformation to a score on a scale ranging from 1 to 100, with a higher score representing better GHS/QoL.^10^ While patient-reported QoL assessments such as EORTC QLQ-C30 GHS/QoL summary scores are informative, they are still primarily used in clinical studies. Interpretation of GHS/QoL scores by both physicians and patients can be challenging in routine cancer care as it is difficult to derive relevance from the scores and determine when they become actionable.^11^ This research addresses this challenge. Giesinger et al.^11^ previously established thresholds of clinical importance for the functioning and symptom scales of the EORTC QLQ-C30 to allow the conversion of absolute scores to prevalence rates for easier use in routine clinical practice. Similarly, to enhance and appreciate the value of the GHS/QoL dataset from the POLARIS study, we sought to translate the EORTC QLQ-C30 overall health (Q29) and QoL (Q30) scores into more meaningful terms for patients and healthcare providers.

## Methods

### Study design

POLARIS (NCT03280303) was a prospective, observational, multi-center, real-world study.

**Figure 1.**
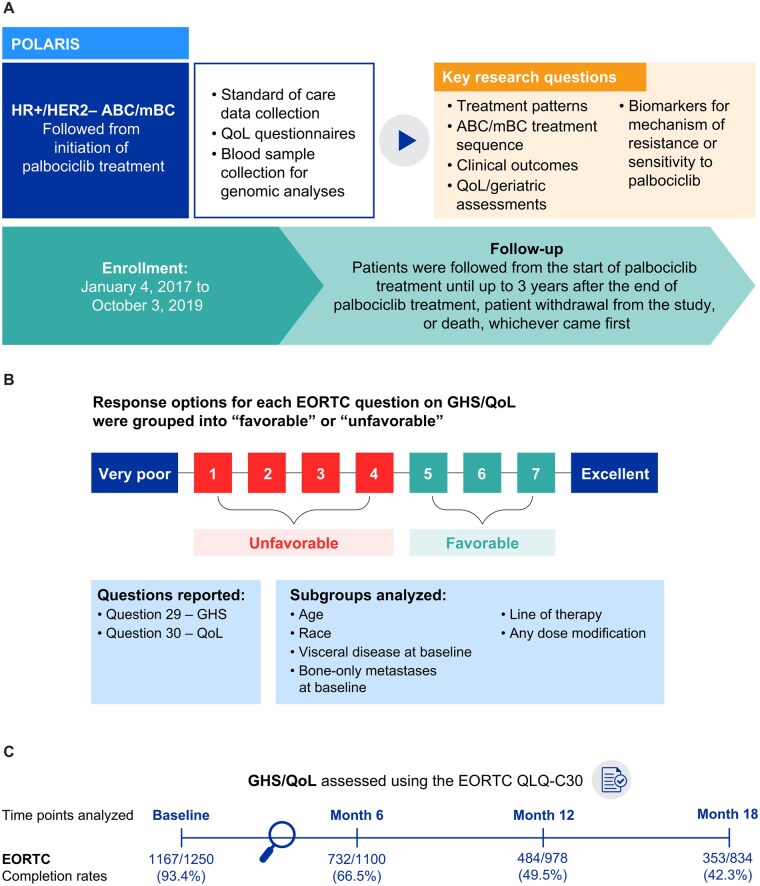
POLARIS (NCT03280303) study design (A), response options on GHS/QoL questions and grouping into favorable and unfavorable categories (B), and EORTC QLQ-C30 completion rates at baseline and months 6, 12, and 18 (C). The POLARIS study included patients treated with a palbociclib-containing regimen given as the first, second, or later line of therapy. In (C), completion rates were calculated according to the number of patients with responses to the GHS/QoL domain at each study time point divided by the total eligible study sample of patients at each time point (ie, *n* = those with continued palbociclib treatment and thus potentially available to answer the questionnaire). Abbreviations: ABC/mBC, advanced or metastatic breast cancer; BIPOC, Black, Indigenous, and People of Color; EORTC QLQ-C30, European Organisation for Research and Treatment of Cancer Quality of Life Questionnaire Core 30; GHS, global health status; HR+/HER2−, hormone receptor-positive/human epidermal growth factor receptor 2–negative; QoL, quality of life.

It was conducted in >100 sites in the US (majority of sites) and Canada according to each site’s routine clinical practice and reviewed and approved by local institutional review boards. Enrollment occurred between January 4, 2017, and October 3, 2019. Written informed consent was obtained from all patients before study enrollment. The POLARIS study design is shown in Figure 1A. The rationale, purpose, and design of the POLARIS study have been reported previously.^8^

### Patient population

Patients ≥18 years of age who had a diagnosis of HR+/HER2− ABC/mBC and received palbociclib (treatment indication determined by a physician) as first-line (1L), second-line (2L), or later line of therapy (LOT) were enrolled in POLARIS. Patients were followed from the start of palbociclib treatment for up to 3 years after the end of treatment, patient study withdrawal, or death, whichever came earliest. Patient characteristics and treatment information were collected by routine clinical assessments performed by treating physicians.

### Patient-reported GHS/QoL

EORTC QLQ-C30 data were collected at baseline, monthly for the first 3 months, and every 3 months thereafter until palbociclib discontinuation. QoL was evaluated using patient-­reported assessment of GHS/QoL on the EORTC QLQ-C30 (version 3) questions: Q29 “How would you rate your overall health during the past week?” and Q30 “How would you rate your overall quality of life during the past week?”.^12^^,^^13^ Response options for Q29 and Q30 that were on a 7-point Likert scale (1 = very poor to 7 = excellent) were categorized into “favorable” (taking a conservative approach of using only responses = 5, 6, or 7) and “unfavorable” (responses of ≤4) responses (Figure 1B). Completion rates of the GHS/QoL domain and the proportions of patients with favorable and unfavorable responses were determined at baseline (ie, date of study enrollment when informed consent was obtained and inclusion/exclusion criteria assessed) and key follow-up time points (months 6, 12, and 18).

### Subgroup analyses

Pre-planned subgroup analyses of the proportions of patients with favorable versus unfavorable responses to Q29 and Q30, separately, were conducted by age (<70 vs ≥70 years), race/ethnicity (Black, Indigenous and People of Color [BIPOC] vs White/not Hispanic or Latino), visceral disease at baseline (yes vs no), bone-only metastases at baseline (yes vs no), LOT (1L vs ≥2L) and any dose modification(s) (yes vs no).

### Statistical analyses

Patient demographic and disease characteristics were summarized descriptively for the overall study population in POLARIS and the per-label population (*n* = 861), consisting of patients with HR+/HER2− ABC/mBC in the study population who were treated as per the US label indication, defined as palbociclib plus an aromatase inhibitor in the 1L setting or palbociclib plus fulvestrant after prior ET in any setting^14^ and are as previously reported.^9^ Descriptive analyses of EORTC QLQ-C30 data were performed on the overall POLARIS study population and were repeated for the per-label population. Response rates to the GHS/QoL domain were calculated according to the number of patients with completed responses to the domain at each study time point (baseline, and months 6, 12, and 18) divided by the number of eligible patients at each time point (ie, those with continued palbociclib treatment and thus potentially available to answer the questionnaire).

For each of the questions, Q29 (GHS) and Q30 (QoL), the proportion of patients with a favorable response for each follow-up time point versus baseline was evaluated using McNemar’s test^15^; with subgroup analyses conducted for patients grouped by type of enrollment site (academic center or community practice) and by ET partner (aromatase inhibitor, fulvestrant, or other ET/no ET). The proportions of patients with favorable and unfavorable responses were compared between the pre-planned subgroups at baseline and months 6, 12, and 18 using Fisher’s exact test.^15^ All comparisons were considered significant at *P *< .05.

## Results

### Study population

Between January 2017 and January 2023, 1250 patients were enrolled in POLARIS and had received ≥1 dose of palbociclib. Of the 123 sites where patients were enrolled, 90 (73.2%) were community practices, 15 (12.2%) were academic centers, 13 (10.6%) were other sites, and details were missing for 5 (4.1%) sites. Patient baseline characteristics are shown in Table 1. Among the study population, median age was 64.0 years, 98.8% were female, 81.8% were White and 11.1% were Black. The median duration from ABC/mBC diagnosis to study enrollment was 1.3 months. At the time of enrollment, 94.9% had metastatic disease, and 5.0% had locally advanced disease. Of those with metastatic disease, 41.7% had visceral disease and 34.1% had bone-only metastases. A majority of patients (72.1%) received palbociclib in the 1L setting (median duration of treatment was 14.2 months); 15.0% and 13.0% of patients received palbociclib as 2L and >2L, respectively (median duration of ≥2L treatment was 9.2 months). Over half (58.2%) of patients received palbociclib with an aromatase inhibitor, 39.4% received fulvestrant, and 2.5% received another ET or no ET.

**Table 1.** Patient baseline demographic and disease characteristics.

Characteristics	Patients (*N* = 1250)
**Age at enrollment, years**	
**Median (range)**	64.0 (22-97)
**Distribution,** *n* **(%)**	
**<50**	193 (15.5)
**50-69**	640 (51.4)
**≥70**	413 (33.1)
**Sex, *n* (%)**	
**Male**	15 (1.2)
**Female**	1235 (98.8)
**Race, *n* (%)**	
**White**	1022 (81.8)
**Black**	139 (11.1)
**Asian**	23 (1.8)
**American Indian or Alaska Native**	8 (0.6)
**Native Hawaiian or other Pacific Islander**	5 (0.4)
**Other**	22 (1.8)
**Not reported/missing**	31 (2.5)
**Ethnicity, *n* (%)**	
**Hispanic or Latino**	106 (8.5)
**Not Hispanic or Latino**	1106 (88.5)
**Not reported/missing**	38 (3.0)
**Disease stage at enrollment, *n* (%)**	
**Locally advanced**	62 (5.0)
**Metastatic**	1186 (94.9)
**Not reported**	2 (0.2)
**Site of distant metastases at mBC diagnosis,** ^a^ ***n* (%)**	
**Visceral disease**	494 (41.7)
**Bone-only**	405 (34.1)
**Bone plus other metastases**	481 (40.6)
**Disposition at enrollment, *n* (%)**	
**Recurrent from earlier stage, stages 0-III**	849 (67.9)
**De novo, stage IV at/near initial diagnosis**	341 (27.3)
**Not reported**	60 (4.8)
**Time from ABC/mBC diagnosis to enrollment, months**	
Median (range)	1.3 (0-248)
**Missing,** *n*	4
**Distribution,** *n* **(%)**	
**≤1 month**	514 (41.3)
**>1-2 months**	245 (19.7)
**>2-6 months**	128 (10.3)
**>6 months**	359 (28.8)
**Line of therapy,** ^b^ *** n* (%)**	
**1L**	901 (72.1)
**2L**	187 (15.0)
**>2L**	162 (13.0)
**ET partner, *n* (%)**	
**Aromatase inhibitor**	727 (58.2)
**Fulvestrant**	492 (39.4)
**Other or no ET**	31 (2.5)

Abbreviations: 1L, first-line; 2L, second-line; >2L, greater than second-line; ABC/mBC, advanced or metastatic breast cancer; ET, endocrine therapy; LOT, line of therapy.

a Among patients with metastatic disease at study enrollment. Visceral disease refers to metastases of the brain, liver, and/or lung/pleura.

b LOT is defined as the number of systemic therapies taken after initial diagnoses of advanced or metastatic breast cancer but before starting palbociclib treatment. First-line patients had no LOT before palbociclib initiation.

### Global health status/quality of life

**Figure 2.**
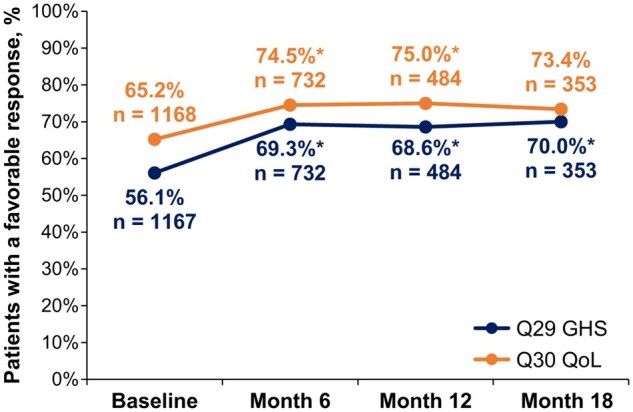
The proportions of patients with a favorable response on EORTC QLQ-C30 Q29 and Q30 at baseline and months 6, 12, and 18. Favorable response rates to Q29 and Q30 were separately calculated according to the number of patients with responses (numeric scores 5**-**7) to the domain at each study time point divided by the total eligible study sample of patients at each time point (ie, *n* = those with continued palbociclib treatment and thus potentially available to answer the questionnaire). ^*^P < .05, versus baseline, which indicates a favorable response was statistically more likely at that time point, from McNemar’s test provided in Table 2. Abbreviations: EORTC QLQ-C30, European Organisation for Research and Treatment of Cancer Quality of Life Questionnaire Core 30; Q, question.

EORTC QLQ-C30 GHS/QoL domain completion rates were 93.4% (*n* = 1167/1250) at baseline, 66.5% (*n* = 732/1100) at month 6, 49.5% (*n* = 484/978) at month 12 and 42.3% (*n* = 353/834) at month 18 (Figure 1C). At baseline, 56.1% of patients had a favorable response to Q29 on GHS and 65.2% to Q30 on QoL. For Q29 (GHS), the proportion of patients with a favorable response significantly increased by ∼13% to 69.3% by month 6, an increase which was maintained at month 12 (68.6%) and month 18 (70.0%; Figure 2). For Q30 (QoL), the proportion of patients with a favorable response significantly increased by ∼9% to 74.5% by month 6, an increase which was also maintained at month 12 (75.0%) and month 18 (73.4%; Figure 2). The proportions of patients with a favorable response to Q29 (GHS) and Q30 (QoL) were consistently higher than those with an unfavorable response across all time points. For Q29, the favorable versus unfavorable response rates were 56.1% versus 43.9% at baseline, 69.3% versus 30.7% at month 6, 68.6% versus 31.4% at month 12, and 70.0% versus 30.0% at month 18. For Q30, the favorable versus unfavorable response rates were 65.2% versus 34.8% at baseline, 74.5% versus 25.5% at month 6, 75.0% versus 25.0% at month 12, and 73.4% versus 26.6% at month 18.

The proportion of patients with a favorable versus unfavorable response was examined at each of the 3 follow-up times relative to baseline. For Q29 and Q30, a favorable response was statistically more likely at each post-baseline time point (6, 12, 18 months) (*P *< .05), except for Q30 at month 18, which was numerically but not statistically more likely than at baseline (*P *= .269) (Figure 2 and Table 2). A stable set of favorable responses was found from month 6 onward. Thus, relative to baseline, these patients had sustained improved QoL over at least an 18-month treatment period. Allowing for differences in sample sizes, we found that the results were generally consistent between patients enrolled at community practices and those enrolled at academic centers (Tables S1 and S2). Of the patients who switched their status from baseline to a particular post-baseline visit (ie, from favorable to unfavorable or from unfavorable to favorable), a higher proportion of them generally gave a favorable response over an unfavorable response at a post-baseline visit. This finding also held true when assessing responses of patients grouped by the endocrine partner (ie, aromatase inhibitor or fulvestrant) received in combination with palbociclib (Tables S3 and S4). The data on palbociclib in combination with other ET/no ET are too sparse for any reliable descriptive assessment (Table S5).

**Table 2.** Change from baseline of response status (favorable vs unfavorable) on EORTC QLQ-C30 Q29 and Q30 at months 6, 12, and 18.^a^

Question 29 (GHS)	Favorable at month 6	Unfavorable at month 6	***P*-value** ^b^
**Favorable at baseline**	338	75	<.001
**Unfavorable at baseline**	147	139	
**Question 30 (QoL)**	Favorable at month 6	Unfavorable at month 6	
**Favorable at baseline**	395	73	<.001
**Unfavorable at baseline**	125	106	
**Question 29 (GHS)**	Favorable at month 12	Unfavorable at month 12	
**Favorable at baseline**	234	57	.015
**Unfavorable at baseline**	86	89	
**Question 30 (QoL)**	Favorable at month 12	Unfavorable at month 12	
**Favorable at baseline**	267	54	.016
**Unfavorable at baseline**	82	63	
**Question 29 (GHS)**	Favorable at month 18	Unfavorable at month 18	
**Favorable at baseline**	168	38	.004
**Unfavorable at baseline**	68	64	
**Question 30 (QoL)**	Favorable at month 18	Unfavorable at month 18	
**Favorable at baseline**	192	44	.269
**Unfavorable at baseline**	55	47	

Abbreviations: EORTC QLQ-C30, European Organisation for Research and Treatment of Cancer Quality of Life Questionnaire Core 30; GHS, global health status; Q, question; QoL, quality of life.

a A cell number in each 2×2 table represents the number of patients who have responded either “favorable” or “unfavorable” at baseline and a post-baseline visit. For example, for Q29 (GHS), 338 patients had a favorable response at baseline and month 6, 75 had a favorable response at baseline and unfavorable response at month 6, 147 had an unfavorable response at baseline and a favorable response at month 6, and 139 had unfavorable responses at both times. Of the patients who switched their status from baseline to a particular post-baseline visit (ie, from favorable to unfavorable or from unfavorable to favorable), a higher proportion of them generally gave a favorable response over an unfavorable response at a post-baseline visit. For instance, of the 222 patients who switched status on Question 29 (GHS), 147 of them (66%) had a favorable response at month 6 compared with 75 of them (34%) who gave an unfavorable response at month 6. Baseline is defined as the last observed measurement before the treatment start date. For each item, only patients who completed the question at both baseline and the time point of interest were included.

b *P-*value is reported from McNemar’s test assessing whether the proportion of patients with a favorable response at the post-baseline time point of interest differs from the proportion of patients with a favorable response at baseline among matched pairs. If the sum of the off-diagonal counts is <25, the exact McNemar test is performed. *P *< .05 was considered statistically significant.

### GHS/QoL in subgroups

**Figure 3.**
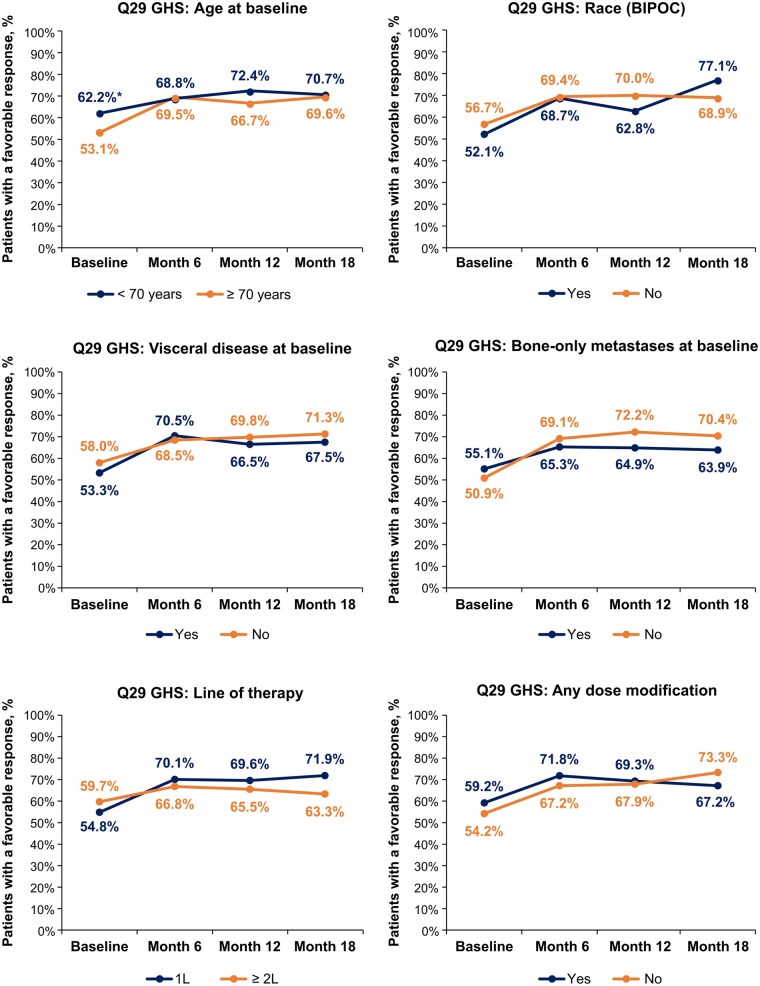
The proportions of patients indicating a favorable response on EORTC QLQ-C30 Q29 across subgroups at baseline and months 6, 12, and 18. Comparative statistical *P-*values were derived using Fisher’s exact test on the association of EORTC QLQ-C30 domains (favorable/unfavorable) with each subgroup at each specific time point. All Fisher’s exact test subgroup comparisons were not considered to be significant (*P* > .05 and, in fact, all *P* > .10), except age at baseline (*P* = .003). ^*^P < .05 indicates statistical significance. Abbreviations: 1L, first-line; 2L, second-line; BIPOC, Black, Indigenous, and People of Color (Yes = BIPOC; No = White/not Hispanic or Latino); EORTC QLQ-C30, European Organisation for Research and Treatment of Cancer Quality of Life Questionnaire Core 30; GHS, global health status; Q, question.

**Figure 4.**
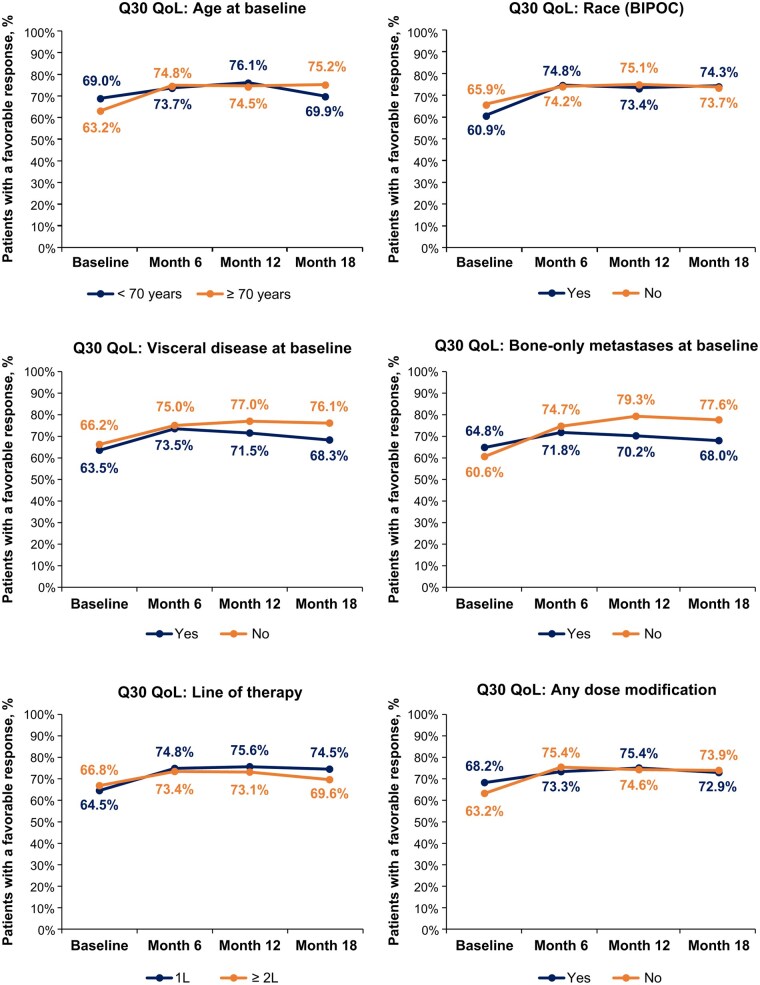
The proportions of patients indicating a favorable response on EORTC QLQ-C30 Q30 across subgroups at baseline and months 6, 12, and 18. Comparative statistical *P-*values were derived using Fisher’s exact test on the association of EORTC QLQ-C30 domains (favorable/unfavorable) with each subgroup at each specific time point. All Fisher’s exact test subgroup comparisons were not considered significant (all *P* > .05). Abbreviations: 1L, first-line; 2L, second-line; BIPOC, Black, Indigenous, and People of Color (Yes = BIPOC; No = White/not Hispanic or Latino); EORTC QLQ-C30, European Organisation for Research and Treatment of Cancer Quality of Life Questionnaire Core 30; Q, question; QoL, quality of life.

In the evaluated subgroups, no significant differences between any subgroups were observed in the proportions of patients with a favorable versus unfavorable response to Q29, except for age at baseline, in which significantly fewer patients with favorable responses were observed among patients with age ≥70 years versus <70 years, but this difference was quickly lost by month 6 (Figure 3). No significant differences were observed in the proportions of patients with a favorable versus unfavorable response to Q30 across any subgroups (Figure 4).

### GHS/QoL in the per-label population

The per-label population included 861 patients (68.9% of the overall POLARIS study population). Among the per-label ­population, 82.7% received palbociclib in the 1L setting (∼10% more than in the overall population), and 8.6% and 8.7% received palbociclib as 2L and >2L, respectively. Patient demographic and disease characteristics were generally consistent with the overall POLARIS population (Table S6). The findings on GHS/QoL in the per-label population aligned closely with that of the overall POLARIS population. For Q29 (GHS), the proportion of patients with a favorable response significantly increased by ∼13% to 69.2% by month 6, an increase which was maintained at month 12 (70.3%) and month 18 (69.8%; Figure S1 [see online supplementary ­material for a color version of this figure]). For Q30 (QoL), the proportion of patients with a favorable response significantly increased by ∼11% to 75.3% by month 6, an increase which was also maintained at month 12 (76.0%) and month 18 (74.4%; ­Figure S1 [see online supplementary material for a color version of this figure]). For Q29 and Q30, a favorable response was statistically more likely at each post-baseline time point (6, 12, 18 months) (*P *< .05), except for Q30 at month 18, which was numerically but not statistically more likely than at baseline (*P *= .123) (Figure S1 [see online supplementary material for a color version of this figure] and Table S7).

### GHS/QoL in per-label subgroups

In the evaluated subgroups, some significant differences were observed in the proportions of patients with a favorable versus unfavorable response to Q29; significantly fewer patients with favorable responses at baseline were found among patients aged ≥70 years versus <70 years and also among those with visceral disease versus those without, but in both cases, these differences were quickly lost by month 6 (Figure S2, see online supplementary material for a color version of this figure). Among the patients who received palbociclib as ≥2L, the proportion with favorable responses to Q29 dropped to 45.2% (14 of 31 patients) at month 18, which was significantly different from the proportion treated with 1L palbociclib (73.1%, 166 of 227) at month 18 (Figure S2, see online supplementary material for a color version of this figure). As with Q29, fewer patients aged ≥70 years had favorable responses to Q30 versus those aged <70 years (Figure S3, see online supplementary material for a color version of this figure). Except for a lower proportion of patients with bone-only metastases at baseline versus those without at month 12 having favorable responses to Q30, no other significant differences were observed in the proportions of patients with a favorable versus unfavorable response between any other per-label subgroups (Figure S3, see online supplementary material for a color version of this figure).

## Discussion

In this analysis of health-related QoL of patients with HR+/HER2– ABC/mBC using patient-reported assessment on the EORTC QLQ-C30 GHS/QoL domain, we have expressed the findings as prevalence of favorable and unfavorable responses to provide a better understanding and an enriched interpretation of the GHS/QoL dataset in the POLARIS study. We found that compared with baseline, in the 6 months after starting palbociclib plus ET, significantly higher proportions of patients continuing on treatment had favorable versus unfavorable GHS and QoL, which were maintained for up to 18 months while patients continued to receive treatment. In the first 6 months of treatment, the proportion of patients with a favorable response on Q29 (GHS) increased by 13% to 69%, and on Q30 (QoL), the proportion increased by 9% to 75%. Similar findings were observed for patients in the per-label population. Overall, these results indicate that patients with HR+/HER2− ABC treated with palbociclib plus ET maintain their QoL for at least 18 months. The simple interpretation on GHS and QoL scores in this study in terms of higher proportion of patients indicating a favorable response over time provides further evidence of the favorable risk–benefit profile of palbociclib plus ET in the treatment of HR+/HER2− ABC.

This translation of the original GHS/QoL data in the POLARIS study now provides prevalence rates of patients with favorable and unfavorable responses and may aid in the interpretation of the originally reported mean scores, which increased from baseline (64.0) an average of 3.5 points after 6 months and was an average of 2.5 points higher than baseline at both months 12 and 18.^9^ These mean changes in the GHS/QoL domain score did not reach the 10-point clinically meaningful threshold.^9^^,^^16^ However, these mean scores on the GHS/QoL domain were higher at months 6 (69.3), 12 (70.1), and 18 (69.9) than that reported as a reference value for the general US population (63.9).^9^^,^^17^

Also notable in the current analysis, no significant differences were observed in the proportions of patients with a favorable versus unfavorable response to Q29 and Q30 for any subgroup (stratified by age, race/ethnicity, visceral disease, bone-only metastases, LOT, and any dose modification) at months 6, 12, and 18 while continuing treatment with palbociclib plus ET. These findings also align with the original GHS/QoL results in the POLARIS study, which show no significant difference in mean GHS/QoL scores at months 6, 12, and 18 across the six subgroups evaluated.^9^ In the per-label population, some differences were observed in GHS/QoL among subgroups. Significantly fewer patients treated with ≥2L palbociclib at month 18 had favorable responses to Q29 GHS than those treated with 1L palbociclib, which was similar for Q30, although this did not reach significance. The number of patients in the ≥2L per-label subgroup (*n* = 31) with responses on the EORTC QLQ-C30 GHS/QoL domain at that time point was low and thus caution is needed in drawing conclusions from this subgroup analysis. These findings could be related to disease progression in these patients; among all patients treated with ≥2L palbociclib in POLARIS, median real-world progression-free survival (PFS) was 13.5 months (95% confidence interval [CI], 10.6-17.1).^18^ Also, in the per-label population, a lesser proportion of patients with baseline bone-only metastases compared with those without had favorable responses to Q30 at month 12. Further research is warranted to confirm these exploratory findings on GHS/QoL differences between certain subgroups in the per-label population.

Because patients with HR+/HER2– ABC/mBC are living longer with their cancer, our study findings showing that GHS/QoL was maintained for most patients treated with palbociclib plus ET while on active treatment is important.^19^^,^^20^ A recently published study in the US, using the Surveillance Epidemiology, and End Results Program (SEER) database, reported that patients diagnosed after the treatment guidelines for cyclin-dependent kinase 4/6 inhibitor use were released (post-2015) had a 10% reduction (vs pre-2015) in the risk of breast cancer-specific death.^20^ The POLARIS study and other real-world studies of patients with HR+/HER2− ABC/mBC have also reported favorable OS rates with palbociclib treatment.^18^^,^^21^^,^^22^ Currently, patients with HR+/HER2– ABC/mBC are treated to delay progression and prolong survival while minimizing the burden of symptoms and treatment-related adverse events.^2^^,^^23^ Surveys of patients with ABC/mBC have consistently found that QoL is equally or more important than OS and PFS in the treatment decision-making process.^3^^,^^24^^,^^25^ As PRO assessments reflect patient perspectives on the management of their disease, it is important for patients to be able to interpret the findings of validated and widely used QoL assessment tools like the EORTC QLQ-C30,^23^ especially as their use increases with electronic device enabled patient-reporting in clinical trials and routine cancer care.^26^ Because abstract numbers, such as metric data on a 0-100 scale, may not mean much for patients, and it is difficult for healthcare providers to explain the meaning of the scores on the EORTC QLQ-C30,^11^ we have translated the absolute mean score findings on the GHS/QoL domain into easier-to-understand proportions of patients with favorable versus unfavorable responses. This may further enhance patient understanding of PRO assessments, which, when used, have been associated with better patient satisfaction, patient-provider communication, and patient-centered care, as well as improved health outcomes in patients with cancer.^23^^,^^26^

The POLARIS study included a large sample of patients (*n* = 1250) with HR+/HER2− ABC/mBC, and there were no exclusions based on age or comorbidities. The patient population was diverse and included male patients (1%), patients who were 70 years of age and older (33%), Black (11%), Asian (2%), and Hispanic or Latino (9%). The large and diverse study population makes the study findings and conclusions more generalizable, with less selection biases. However, the heterogeneous, less-selective patient population may increase the difficulty of interpreting GHS/QoL outcomes.^8^

As seen in other real-world studies in the advanced cancers setting,^27^^,^^28^ the proportion of patients completing the GHS/QoL domain on the EORTC QLQ-C30 decreased over time. However, as reported previously,^9^ characteristics were relatively similar among patients who completed the GHS/QoL domain at baseline and the other analyzed time points, suggesting no clear evidence of subpopulation selection bias. In the POLARIS study, negative events experienced by patients (eg, disease progression [60%], treatment toxicities [10%], death [42%], etc.) contributed to the attrition of completed questionnaires.^9^ There was additionally an impact on completion of questionnaires during the COVID-19 pandemic; approximately a third of the POLARIS study sites experienced a decrease in responsiveness to correspondence and delays in data entry.^29^ In a sensitivity analysis of patients who provided continual completed questionnaire submissions, GHS/QoL domain mean values over time were generally consistent with the main analysis, further indicating that attrition might not have introduced sizable bias.^9^ By design, the POLARIS study characterized the QoL of patients actively receiving palbociclib treatment; thus, as patients discontinued palbociclib over the course of the study, they no longer completed QoL assessments.

As a real-world observational study, incomplete and inaccurate data may have been present in the POLARIS dataset, and causality cannot be confirmed between treatment and outcomes. Also, it was beyond the scope of the analyses to examine the impact of treatment discontinuation, disease progression, or other factors on GHS/QoL. Lastly, other differences in patient and disease characteristics were not adjusted for in the comparative subgroup analyses.

## Conclusions

The proportions of patients with HR+/HER2− ABC/mBC indicating a favorable response on the EORTC QLQ-C30 questions on GHS and QoL increased early on after the start of palbociclib treatment and were preserved through month 18 across the overall POLARIS study population and most evaluated subgroups. The simple interpretation on GHS and QoL scores given here is intended to enhance their meaning for the benefit of patients and other stakeholders. Such longitudinal PRO assessments are valuable to clinicians and patients, but only when the assessments are understood and used to inform care management. It will be important that future research is directed toward gaining a better understanding of patient perspectives on how PRO assessment tools improve their care, especially as their use increases for routine patient monitoring with electronic devices.

## Supplementary Material

oyaf281_Supplementary_Data

## Data Availability

Upon request, and subject to review, Pfizer will provide the data that support the findings of this study. Subject to certain criteria, conditions, and exceptions, Pfizer may also provide access to the related individual de-identified participant data. See https://www.pfizer.com/science/clinical-trials/trial-data-and-results for more information.

## References

[1] Loeser A , Kim JS, Peppercorn J, et al The right dose: results of a patient advocate-led survey of individuals with metastatic breast cancer regarding treatment-related side effects and views about dosage assessment to optimize quality of life. JCO Oncol Pract. 2024;20:972-983. 10.1200/op.23.00539 38518184

[2] Hillebrand LE , Söling U, Marschner N. Significance of patient-reported outcomes for metastatic breast cancer patients. Oncol Res Treat. 2022;45:423-429. 10.1159/000521826 34999590

[3] Reinisch M , Marschner N, Otto T, Korfel A, Stoffregen C, Wöckel A. Patient preferences: results of a German adaptive choice-based conjoint analysis (market research study sponsored by Eli Lilly and Company) in patients on palliative treatment for advanced breast cancer. Breast Care (Basel). 2021;16:491-499. 10.1159/000513139 34720809 PMC8543321

[4] U.S. Department of Health and Human Services: Food and Drug Administration. Submitting patient-reported outcome data in cancer clinical trials: guidance for industry. Technical Specifications Document. Accessed July 31, 2024. https://www.fda.gov/regulatory-information/search-fda-guidance-documents/submitting-patient-reported-outcome-data-cancer-clinical-trials

[5] Kluetz PG , Chingos DT, Basch EM, Mitchell SA. Patient-reported outcomes in cancer clinical trials: measuring symptomatic adverse events with the National Cancer Institute’s Patient-Reported Outcomes Version of the Common Terminology Criteria for Adverse Events (PRO-CTCAE). Am Soc Clin Oncol Educ Book. 2016;35:67-73. 10.1200/edbk_159514 27249687

[6] Rugo HS , Dieras V, Gelmon KA, et al Impact of palbociclib plus letrozole on patient-reported health-related quality of life: results from the PALOMA-2 trial. Ann Oncol. 2018;29:888-894. 10.1093/annonc/mdy012 29360932 PMC5913649

[7] Harbeck N , Iyer S, Turner N, et al Quality of life with palbociclib plus fulvestrant in previously treated hormone receptor-positive, HER2-negative metastatic breast cancer: patient-reported outcomes from the PALOMA-3 trial. Ann Oncol. 2016;27:1047-1054. 10.1093/annonc/mdw139 27029704 PMC4880065

[8] Tripathy D , Blum JL, Rocque GB, et al POLARIS: a prospective, multicenter, noninterventional study assessing palbociclib in hormone receptor-positive advanced breast cancer. Future Oncol. 2020;16:2475-2485. 10.2217/fon-2020-0573 32787449

[9] Rocque G , Blum JL, Ji Y, et al Real-world quality-of-life of patients with HR+/HER2− advanced breast cancer treated with palbociclib plus endocrine therapy: EORTC QLQ-C30 results from POLARIS. Breast Cancer Res Treat. 2025;209:613-627. 10.1007/s10549-024-07524-2 39581892 PMC11785676

[10] Fayers P , Machin D. Quality of Life: The Assessment, Analysis and Reporting of Patient-Reported Outcomes. 3rd ed. Wiley-Blackwell; 2016.

[11] Giesinger JM , Loth FLC, Aaronson NK, et al; EORTC Quality of Life Group. Thresholds for clinical importance were established to improve interpretation of the EORTC QLQ-C30 in clinical practice and research. J Clin Epidemiol. 2020;118:1-8. 10.1016/j.jclinepi.2019.10.003 31639445

[12] Aaronson NK , Ahmedzai S, Bergman B, et al The European Organization for Research and Treatment of Cancer QLQ-C30: a quality-of-life instrument for use in international clinical trials in oncology. J Natl Cancer Inst. 1993;85:365-376. 10.1093/jnci/85.5.365 8433390

[13] EORTC Quality of Life Group. EORTC QLQ-C30 (version 3). Accessed June 5, 2024. https://www.eortc.org/app/uploads/sites/2/2018/08/Specimen-QLQ-C30-English.pdf

[14] Pfizer Labs. IBRANCE (palbociclib) - Highlights of prescribing information. Accessed March 29, 2024. https://www.accessdata.fda.gov/drugsatfda_docs/label/2019/207103s008lbl.pdf

[15] Rosner B. Fundamentals of Biostatistics. 8th ed. Cengage Learning; 2015.

[16] Osoba D , Rodrigues G, Myles J, Zee B, Pater J. Interpreting the significance of changes in health-related quality-of-life scores. J Clin Oncol. 1998;16:139-144. 10.1200/jco.1998.16.1.139 9440735

[17] Nolte S , Liegl G, Petersen MA, et al; EORTC Quality of Life Group. General population normative data for the EORTC QLQ-C30 health-related quality of life questionnaire based on 15,386 persons across 13 European countries, Canada and the Unites States. Eur J Cancer. 2019;107:153-163. 10.1016/j.ejca.2018.11.024 30576971

[18] Tripathy D , Blum JL, Karuturi MS, et al Real-world effectiveness of palbociclib plus endocrine therapy in HR+/HER2– advanced breast cancer: final results from the POLARIS trial. Oncologist. 2025;30:oyae291. 10.1093/oncolo/oyae291 39475418 PMC12311288

[19] Meegdes M , Geurts SME, Erdkamp FLG, et al Real-world time trends in overall survival, treatments and patient characteristics in HR+/HER2– metastatic breast cancer: an observational study of the SONABRE Registry. Lancet Reg Health Eur. 2023;26:100573. 10.1016/j.lanepe.2022.100573 36895447 PMC9989628

[20] Brufsky A , Kwan ML, Sandin R, et al Trends in HR+ metastatic breast cancer survival before and after CDK4/6 inhibitor introduction in the United States: a SEER registry analysis of patients with HER2- and HER2+ metastatic breast cancer. Breast Cancer Res Treat. 2024;208:223-235. 10.1007/s10549-024-07469-6 PMC11455714 39177933

[21] Rugo HS , Brufsky A, Liu X, et al Real-world study of overall survival with palbociclib plus aromatase inhibitor in HR+/HER2- metastatic breast cancer. NPJ Breast Cancer. 2022;8:114. 10.1038/s41523-022-00479-x 36220852 PMC9553912

[22] Taylor-Stokes G , Mitra D, Waller J, Gibson K, Milligan G, Iyer S. Treatment patterns and clinical outcomes among patients receiving palbociclib in combination with an aromatase inhibitor or fulvestrant for HR+/HER2-negative advanced/metastatic breast cancer in real-world settings in the US: results from the IRIS study. Breast. 2019;43:22-27. 10.1016/j.breast.2018.10.009 30391832

[23] Clarijs ME , Thurell J, Kühn F, et al Measuring quality of life using patient-reported outcomes in real-world metastatic breast cancer patients: the need for a standardized approach. Cancers (Basel). 2021;13:2308. 10.3390/cancers13102308 PMC8151772 34065805

[24] Bland KA , Mustafa R, McTaggart-Cowan H. Patient preferences in metastatic breast cancer care: a scoping review. Cancers (Basel). 2023;15:4331. 10.3390/cancers15174331 37686607 PMC10486914

[25] Mertz S , Benjamin C, Girvalaki C, et al Progression-free survival and quality of life in metastatic breast cancer: the patient perspective. Breast. 2022;65:84-90. 10.1016/j.breast.2022.07.006 35870420 PMC9307669

[26] van Egdom LSE , Oemrawsingh A, Verweij LM, et al Implementing patient-reported outcome measures in clinical breast cancer care: a systematic review. Value Health. 2019;22:1197-1226. 10.1016/j.jval.2019.04.1927 31563263

[27] Fang FM , Tsai WL, Chien CY, et al Changing quality of life in patients with advanced head and neck cancer after primary radiotherapy or chemoradiation. Oncology. 2005;68:405-413. 10.1159/000086982 16020970

[28] Ramsey I , de Rooij BH, Mols F, et al Cancer survivors who fully participate in the PROFILES registry have better health-related quality of life than those who drop out. J Cancer Surviv. 2019;13:829-839. 10.1007/s11764-019-00793-7 31493162 PMC6881419

[29] Tripathy D , Blum JL, Rocque G, et alAbstract SS2-10: Impact of COVID-19 on study sites: survey analysis from the noninterventional POLARIS study. Cancer Res. 2021;81:SS2-10. 10.1158/1538-7445.Sabcs20-ss2-10

