# Supplementary material for: Enhancing the interpretation of real-world quality of life in patients with hormone receptor-positive/human epidermal growth factor receptor 2-negative advanced breast cancer enrolled in the POLARIS study
Source: Oncologist. 2025 Sep 15;30(10):oyaf281. doi: 10.1093/oncolo/oyaf281 (PMC12596709; doi:10.1093/oncolo/oyaf281)
Supplement: oyaf281_Supplementary_Data [file oyaf281_supplementary_data.docx]

**Supplemental material**

**Enhancing the interpretation of real-world quality of life in patients with hormone receptor-positive/human epidermal growth factor 2-negative advanced breast cancer enrolled in the POLARIS study**

**Authors:** Gabrielle Rocque,^1^ Joanne L. Blum,^2^ Yan Ji,^3^ Timothy Pluard,^4^ John Migas,^5^ Shailendra Lakhanpal,^6^ Erin Jepsen,^7^ Eric Gauthier,^8^ Yao Wang,^9^ Monica Z. Montelongo,^10^ Joseph C. Cappelleri,^11^ Connie Chen,^12^ Meghan S. Karuturi,^13^ Debu Tripathy^13^

**Affiliations:** ^1^University of Alabama at Birmingham, Birmingham, AL, USA; ^2^Baylor-Sammons Cancer Center, Texas Oncology, US Oncology, Dallas, TX, USA; ^3^Health Partners Institute, St. Paul, MN, USA; ^4^Saint Luke’s Cancer Institute, Kansas City, MO, USA; ^5^Mid-Illinois Hematology & Oncology Associates Ltd., Normal, IL, USA; ^6^Saint Vincent’s Birmingham^,^ Birmingham, AL, USA; ^7^Novant Health, Winston-Salem, NC, USA; ^8^Pfizer Inc, San Francisco, CA, USA; ^9^Pfizer Inc, La Jolla, CA, USA; ^10^ICON plc, Blue Bell, PA, USA; ^11^Pfizer Inc, Groton, CT, USA; ^12^Pfizer Inc, New York, NY, USA; ^13^The University of Texas MD Anderson Cancer Center, Houston, TX, USA.

**Corresponding author:**

Gabrielle Rocque, MD

University of Alabama at Birmingham

2000 6th Ave S

Birmingham, AL 35233

Email: grocque@uabmc.edu

**Supplementary Table S1 Community practices: Change from baseline of response status (favorable vs unfavorable) on EORTC QLQ–C30 Q29 and Q30 at months 6, 12, and 18^a^**

| **Question 29 (GHS)** | Favorable at month 6 | Unfavorable at month 6 | ***P* value^b^** |
| --- | --- | --- | --- |
| Favorable at baseline | 263 | 59 | < 0.001 |
| Unfavorable at baseline | 114 | 105 |  |
| **Question 30 (QoL)** | Favorable at month 6 | Unfavorable at month 6 |  |
| Favorable at baseline | 311 | 57 | 0.010 |
| Unfavorable at baseline | 88 | 85 |  |
| **Question 29 (GHS)** | Favorable at month 12 | Unfavorable at month 12 |  |
| Favorable at baseline | 188 | 42 | 0.075 |
| Unfavorable at baseline | 60 | 70 |  |
| **Question 30 (QoL)** | Favorable at month 12 | Unfavorable at month 12 |  |
| Favorable at baseline | 214 | 40 | 0.069 |
| Unfavorable at baseline | 58 | 48 |  |
| **Question 29 (GHS)** | Favorable at month 18 | Unfavorable at month 18 |  |
| Favorable at baseline | 126 | 29 | 0.008 |
| Unfavorable at baseline | 53 | 53 |  |
| **Question 30 (QoL)** | Favorable at month 18 | Unfavorable at month 18 |  |
| Favorable at baseline | 147 | 34 | 0.486 |
| Unfavorable at baseline | 40 | 40 |  |

*EORTC QLQ–C30* European Organisation for Research and Treatment of Cancer Quality of Life Questionnaire Core 30, *GHS* global health status, *Q* question, *QoL* quality of life

^a^ A cell number in each 2x2 table represents the number of patients who have responded either “favorable” or “unfavorable” at baseline and a post-baseline visit. For example, for Q29 (GHS), 263 patients had a favorable response at baseline and month 6, 59 had a favorable response at baseline and unfavorable response at month 6, 114 had an unfavorable response at baseline and a favorable response at month 6, and 105 had unfavorable responses at both times. Of the patients who switched their status from baseline to a particular post-baseline visit (i.e. from favorable to unfavorable or from unfavorable to favorable), a higher proportion of them generally gave a favorable response over an unfavorable response at a post-baseline visit. For instance, of the 173 patients who switched status on Question 29 (GHS), 114 of them (66%) had a favorable response at month 6 compared with 59 of them (34%) who gave an unfavorable response at month 6. Baseline is defined as the last observed measurement before the treatment start date. For each item, only patients who completed the question at both baseline and the time point of interest were included.

^b^ *P* value is reported from McNemar’s test assessing whether the proportion of patients with a favorable response at the post-baseline time point of interest differs from the proportion of patients with a favorable response at baseline among matched pairs. If the sum of the off-diagonal counts is < 25, the exact McNemar test is performed. *P* < 0.05 was considered statistically significant.

**Supplementary Table S2 Academic centers: Change from baseline of response status (favorable vs unfavorable) on EORTC QLQ–C30 Q29 and Q30 at months 6, 12, and 18^a^**

| **Question 29 (GHS)** | Favorable at month 6 | Unfavorable at month 6 | ***P* value^b^** |
| --- | --- | --- | --- |
| Favorable at baseline | 39 | 7 | 0.263 |
| Unfavorable at baseline | 13 | 16 |  |
| **Question 30 (QoL)** | Favorable at month 6 | Unfavorable at month 6 |  |
| Favorable at baseline | 43 | 8 | 0.210 |
| Unfavorable at baseline | 15 | 9 |  |
| **Question 29 (GHS)** | Favorable at month 12 | Unfavorable at month 12 |  |
| Favorable at baseline | 22 | 6 | 0.078 |
| Unfavorable at baseline | 15 | 7 |  |
| **Question 30 (QoL)** | Favorable at month 12 | Unfavorable at month 12 |  |
| Favorable at baseline | 24 | 7 | 0.134 |
| Unfavorable at baseline | 15 | 4 |  |
| **Question 29 (GHS)** | Favorable at month 18 | Unfavorable at month 18 |  |
| Favorable at baseline | 16 | 8 | 1.000 |
| Unfavorable at baseline | 8 | 5 |  |
| **Question 30 (QoL)** | Favorable at month 18 | Unfavorable at month 18 |  |
| Favorable at baseline | 19 | 7 | 1.000 |
| Unfavorable at baseline | 8 | 3 |  |

*EORTC QLQ–C30* European Organisation for Research and Treatment of Cancer Quality of Life Questionnaire Core 30, *GHS* global health status, *Q* question, *QoL* quality of life

^a^ A cell number in each 2x2 table represents the number of patients who have responded either “favorable” or “unfavorable” at baseline and a post-baseline visit. For example, for Q29 (GHS), 39 patients had a favorable response at baseline and month 6, 7 had a favorable response at baseline and unfavorable response at month 6, 13 had an unfavorable response at baseline and a favorable response at month 6, and 16 had unfavorable responses at both times. Of the patients who switched their status from baseline to a particular post-baseline visit (i.e. from favorable to unfavorable or from unfavorable to favorable), a higher proportion of them generally gave a favorable response over an unfavorable response at a post-baseline visit. For instance, of the 20 patients who switched status on Question 29 (GHS), 13 of them (65%) had a favorable response at month 6 compared with 7 of them (35%) who gave an unfavorable response at month 6. Baseline is defined as the last observed measurement before the treatment start date. For each item, only patients who completed the question at both baseline and the time point of interest were included.

^b^ *P* value is reported from McNemar’s test assessing whether the proportion of patients with a favorable response at the post-baseline time point of interest differs from the proportion of patients with a favorable response at baseline among matched pairs. If the sum of the off-diagonal counts is < 25, the exact McNemar test is performed. *P* < 0.05 was considered statistically significant.

**Supplementary Table S3 Palbociclib + aromatase inhibitor: Change from baseline of response status (favorable vs unfavorable) on EORTC QLQ–C30 Q29 and Q30 at months 6, 12, and 18^a^**

| **Question 29 (GHS)** | Favorable at month 6 | Unfavorable at month 6 | ***P* value^b^** |
| --- | --- | --- | --- |
| Favorable at baseline | 207 | 46 | < 0.001 |
| Unfavorable at baseline | 88 | 70 |  |
| **Question 30 (QoL)** | Favorable at month 6 | Unfavorable at month 6 |  |
| Favorable at baseline | 234 | 42 | < 0.001 |
| Unfavorable at baseline | 81 | 54 |  |
| **Question 29 (GHS)** | Favorable at month 12 | Unfavorable at month 12 |  |
| Favorable at baseline | 151 | 36 | 0.270 |
| Unfavorable at baseline | 46 | 50 |  |
| **Question 30 (QoL)** | Favorable at month 12 | Unfavorable at month 12 |  |
| Favorable at baseline | 164 | 32 | 0.059 |
| Unfavorable at baseline | 49 | 38 |  |
| **Question 29 (GHS)** | Favorable at month 18 | Unfavorable at month 18 |  |
| Favorable at baseline | 119 | 20 | 0.010 |
| Unfavorable at baseline | 40 | 35 |  |
| **Question 30 (QoL)** | Favorable at month 18 | Unfavorable at month 18 |  |
| Favorable at baseline | 128 | 24 | 0.345 |
| Unfavorable at baseline | 31 | 31 |  |

*EORTC QLQ–C30* European Organisation for Research and Treatment of Cancer Quality of Life Questionnaire Core 30, *GHS* global health status, *Q* question, *QoL* quality of life

^a^ A cell number in each 2x2 table represents the number of patients who have responded either “favorable” or “unfavorable” at baseline and a post-baseline visit. For example, for Q29 (GHS), 207 patients had a favorable response at baseline and month 6, 46 had a favorable response at baseline and unfavorable response at month 6, 88 had an unfavorable response at baseline and a favorable response at month 6, and 70 had unfavorable responses at both times. Of the patients who switched their status from baseline to a particular post-baseline visit (i.e. from favorable to unfavorable or from unfavorable to favorable), a higher proportion of them generally gave a favorable response over an unfavorable response at a post-baseline visit. For instance, of the 134 patients who switched status on Question 29 (GHS), 88 of them (66%) had a favorable response at month 6 compared with 46 of them (34%) who gave an unfavorable response at month 6. Baseline is defined as the last observed measurement before the treatment start date. For each item, only patients who completed the question at both baseline and the time point of interest were included.

^b^ *P* value is reported from McNemar’s test assessing whether the proportion of patients with a favorable response at the post-baseline time point of interest differs from the proportion of patients with a favorable response at baseline among matched pairs. If the sum of the off-diagonal counts is < 25, the exact McNemar test is performed. *P* < 0.05 was considered statistically significant.

**Supplementary Table S4 Palbociclib + fulvestrant: Change from baseline of response status (favorable vs unfavorable) on EORTC QLQ–C30 Q29 and Q30 at months 6, 12, and 18^a^**

| **Question 29 (GHS)** | Favorable at month 6 | Unfavorable at month 6 | ***P* value^b^** |
| --- | --- | --- | --- |
| Favorable at baseline | 121 | 26 | < 0.001 |
| Unfavorable at baseline | 56 | 69 |  |
| **Question 30 (QoL)** | Favorable at month 6 | Unfavorable at month 6 |  |
| Favorable at baseline | 149 | 29 | 0.123 |
| Unfavorable at baseline | 42 | 52 |  |
| **Question 29 (GHS)** | Favorable at month 12 | Unfavorable at month 12 |  |
| Favorable at baseline | 80 | 20 | 0.013 |
| Unfavorable at baseline | 39 | 39 |  |
| **Question 30 (QoL)** | Favorable at month 12 | Unfavorable at month 12 |  |
| Favorable at baseline | 98 | 22 | 0.138 |
| Unfavorable at baseline | 33 | 25 |  |
| **Question 29 (GHS)** | Favorable at month 18 | Unfavorable at month 18 |  |
| Favorable at baseline | 45 | 15 | 0.086 |
| Unfavorable at baseline | 26 | 29 |  |
| **Question 30 (QoL)** | Favorable at month 18 | Unfavorable at month 18 |  |
| Favorable at baseline | 58 | 17 | 0.274 |
| Unfavorable at baseline | 24 | 16 |  |

*EORTC QLQ–C30* European Organisation for Research and Treatment of Cancer Quality of Life Questionnaire Core 30, *GHS* global health status, *Q* question, *QoL* quality of life

^a^ A cell number in each 2x2 table represents the number of patients who have responded either “favorable” or “unfavorable” at baseline and a post-baseline visit. For example, for Q29 (GHS), 121 patients had a favorable response at baseline and month 6, 26 had a favorable response at baseline and unfavorable response at month 6, 56 had an unfavorable response at baseline and a favorable response at month 6, and 69 had unfavorable responses at both times. Of the patients who switched their status from baseline to a particular post-baseline visit (i.e. from favorable to unfavorable or from unfavorable to favorable), a higher proportion of them generally gave a favorable response over an unfavorable response at a post-baseline visit. For instance, of the 82 patients who switched status on Question 29 (GHS), 56 of them (68%) had a favorable response at month 6 compared with 26 of them (32%) who gave an unfavorable response at month 6. Baseline is defined as the last observed measurement before the treatment start date. For each item, only patients who completed the question at both baseline and the time point of interest were included.

^b^ *P* value is reported from McNemar’s test assessing whether the proportion of patients with a favorable response at the post-baseline time point of interest differs from the proportion of patients with a favorable response at baseline among matched pairs. If the sum of the off-diagonal counts is < 25, the exact McNemar test is performed. *P* < 0.05 was considered statistically significant.

**Supplementary Table S5 Palbociclib + other ET/no ET: Change from baseline of response status (favorable vs unfavorable) on EORTC QLQ–C30 Q29 and Q30 at months 6, 12, and 18^a^**

| **Question 29 (GHS)** | Favorable at month 6 | Unfavorable at month 6 | ***P* value^b^** |
| --- | --- | --- | --- |
| Favorable at baseline | 10 | 3 | 1.000 |
| Unfavorable at baseline | 3 | 0 |  |
| **Question 30 (QoL)** | Favorable at month 6 | Unfavorable at month 6 |  |
| Favorable at baseline | 12 | 2 | 1.000 |
| Unfavorable at baseline | 2 | 0 |  |
| **Question 29 (GHS)** | Favorable at month 12 | Unfavorable at month 12 |  |
| Favorable at baseline | 3 | 1 | 1.000 |
| Unfavorable at baseline | 1 | 0 |  |
| **Question 30 (QoL)** | Favorable at month 12 | Unfavorable at month 12 |  |
| Favorable at baseline | 5 | 0 | NE |
| Unfavorable at baseline | 0 | 0 |  |
| **Question 29 (GHS)** | Favorable at month 18 | Unfavorable at month 18 |  |
| Favorable at baseline | 4 | 3 | 1.000 |
| Unfavorable at baseline | 2 | 0 |  |
| **Question 30 (QoL)** | Favorable at month 18 | Unfavorable at month 18 |  |
| Favorable at baseline | 6 | 3 | 0.250 |
| Unfavorable at baseline | 0 | 0 |  |

*EORTC QLQ–C30* European Organisation for Research and Treatment of Cancer Quality of Life Questionnaire Core 30, *ET*, endocrine therapy, *GHS* global health status, *NE* not estimable, *Q* question, *QoL* quality of life

^a^ A cell number in each 2x2 table represents the number of patients who have responded either “favorable” or “unfavorable” at baseline and a post-baseline visit. For example, for Q29 (GHS), 10 patients had a favorable response at baseline and month 6, 3 had a favorable response at baseline and unfavorable response at month 6, 3 had an unfavorable response at baseline and a favorable response at month 6, and 0 had unfavorable responses at both times. Baseline is defined as the last observed measurement before the treatment start date. For each item, only patients who completed the question at both baseline and the time point of interest were included. Caution: The data are too sparse for a reliable descriptive assessment.

^b^ *P* value is reported from McNemar’s test assessing whether the proportion of patients with a favorable response at the post-baseline time point of interest differs from the proportion of patients with a favorable response at baseline among matched pairs. If the sum of the off-diagonal counts is < 25, the exact McNemar test is performed. *P* < 0.05 was considered statistically significant.

Supplementary Table S6 Per-label population: Patient baseline demographic and disease characteristics

| **Characteristics** | **Patients (N = 861)** |
| --- | --- |
| **Age at enrollment, years** | |
| Median (range) | 64.0 (22–97) |
| Distribution, n (%) | |
| < 50 | 112 (13.0) |
| 50–69 | 455 (52.9) |
| ≥ 70 | 293 (34.1) |
| **Sex, n (%)** | |
| Male | 12 (1.4) |
| Female | 849 (98.6) |
| **Race, n (%)** | |
| White | 709 (82.3) |
| Black | 99 (11.5) |
| Asian | 11 (1.3) |
| American Indian or Alaska Native | 6 (0.7) |
| Native Hawaiian or other Pacific Islander | 3 (0.3) |
| Other | 15 (1.7) |
| Not reported/missing | 18 (2.1) |
| **Ethnicity, n (%)** | |
| Hispanic or Latino | 61 (7.1) |
| Not Hispanic or Latino | 775 (90.0) |
| Not reported/missing | 25 (2.9) |
| **Disease stage at enrollment, n (%)** | |
| Locally advanced | 37 (4.3) |
| Metastatic | 822 (95.5) |
| Not reported | 2 (0.2) |
| **Site of distant metastases at mBC diagnosis,^a^ n (%)** |  |
| Visceral disease | 331 (40.3) |
| Bone-only | 299 (36.4) |
| Bone plus other metastases | 331 (40.3) |
| **Disposition at enrollment, n (%)** | |
| Recurrent from earlier stage, stages 0–III | 589 (68.4) |
| De novo, stage IV at/near initial diagnosis | 237 (27.5) |
| Not reported | 35 (4.1) |
| **Time from ABC/mBC diagnosis to enrollment, months** | |
| Median (range) | 1.1 (0–193) |
| Missing, n | 4 |
| Distribution, n (%) | |
| ≤ 1 month | 406 (47.4) |
| > 1–2 months | 195 (22.8) |
| > 2–6 months | 79 (9.2) |
| > 6 months | 177 (20.7) |
| **Line of therapy,^b^ n (%)** | |
| 1L | 712 (82.7) |
| 2L | 74 (8.6) |
| > 2L | 75 (8.7) |
| **ET partner, n (%)** |  |
| Aromatase inhibitor | 533 (61.9) |
| Fulvestrant | 328 (38.1) |

*1L* first-line, *2L* second-line, *> 2L* greater than second-line, *ABC/mBC* advanced or metastatic breast cancer, *ET* endocrine therapy, *LOT* line of therapy

^a^ Among patients with metastatic disease at study enrollment. Visceral disease refers to metastases of the brain, liver, and/or lung/pleura

^b^ LOT is defined as the number of systemic therapies taken after initial diagnoses of advanced or metastatic breast cancer but before starting palbociclib treatment. First-line patients had no LOT before palbociclib initiation

**Supplementary Fig. S1 Per-label population: Proportions of patients with a favorable response on EORTC QLQ–C30 Q29 and Q30 at baseline and months 6, 12, and 18**


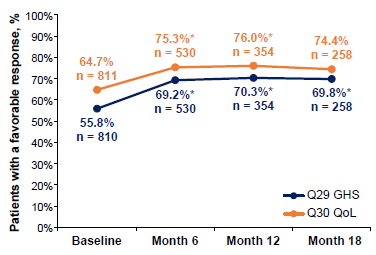


Favorable response rates to Q29 and Q30 were separately calculated according to the number of patients with responses (numeric scores 5–7) to the domain at each study time point divided by the total eligible study sample of patients at each time point (i.e. n = those with continued palbociclib treatment and thus potentially available to answer the questionnaire). * *P* < 0.05, versus baseline, which indicates a favorable response was statistically more likely at that time point, from McNemar’s test provided in Online Resource 3.

*EORTC QLQ–C30* European Organisation for Research and Treatment of Cancer Quality of Life Questionnaire Core 30, *GHS* global health status, *Q* question, *QoL* quality of life

**Supplementary Table S7 Per-label population: Change from baseline of response status (favorable vs unfavorable) on EORTC QLQ–C30 Q29 and Q30 at months 6, 12, and 18^a^**

| **Question 29 (GHS)** | Favorable at month 6 | Unfavorable at month 6 | ***P* value^b^** |
| --- | --- | --- | --- |
| Favorable at baseline | 242 | 49 | < 0.001 |
| Unfavorable at baseline | 113 | 107 |  |
| **Question 30 (QoL)** | Favorable at month 6 | Unfavorable at month 6 |  |
| Favorable at baseline | 284 | 47 | < 0.001 |
| Unfavorable at baseline | 101 | 79 |  |
| **Question 29 (GHS)** | Favorable at month 12 | Unfavorable at month 12 |  |
| Favorable at baseline | 175 | 38 | 0.003 |
| Unfavorable at baseline | 69 | 63 |  |
| **Question 30 (QoL)** | Favorable at month 12 | Unfavorable at month 12 |  |
| Favorable at baseline | 199 | 35 | 0.004 |
| Unfavorable at baseline | 64 | 47 |  |
| **Question 29 (GHS)** | Favorable at month 18 | Unfavorable at month 18 |  |
| Favorable at baseline | 127 | 24 | 0.009 |
| Unfavorable at baseline | 46 | 52 |  |
| **Question 30 (QoL)** | Favorable at month 18 | Unfavorable at month 18 |  |
| Favorable at baseline | 142 | 29 | 0.123 |
| Unfavorable at baseline | 42 | 36 |  |

*EORTC QLQ–C30* European Organisation for Research and Treatment of Cancer Quality of Life Questionnaire Core 30, *GHS* global health status, *Q* question, *QoL* quality of life

^a^ A cell number in each 2x2 table represents the number of patients who have responded either “favorable” or “unfavorable” at baseline and a post-baseline visit. For example, for Q29 (GHS), 242 patients had a favorable response at baseline and month 6, 49 had a favorable response at baseline and unfavorable response at month 6, 113 had an unfavorable response at baseline and a favorable response at month 6, and 107 had unfavorable responses at both times. Of the patients who switched their status from baseline to a particular post-baseline visit (i.e. from favorable to unfavorable or from unfavorable to favorable), a higher proportion of them generally gave a favorable response over an unfavorable response at a post-baseline visit. For instance, of the 162 patients who switched status on Question 29 (GHS), 113 of them (70%) had a favorable response at month 6 compared with 49 of them (30%) who gave an unfavorable response at month 6. Baseline is defined as the last observed measurement before the treatment start date. For each item, only patients who completed the question at both baseline and the time point of interest were included.

^b^ *P* value is reported from McNemar’s test assessing whether the proportion of patients with a favorable response at the post-baseline time point of interest differs from the proportion of patients with a favorable response at baseline among matched pairs. If the sum of the off-diagonal counts is < 25, the exact McNemar test is performed. *P* < 0.05 was considered statistically significant.

**Supplementary Fig. S2 Per-label population: Proportions of patients indicating a favorable response on EORTC QLQ–C30 Q29 (GHS) across subgroups at baseline and months 6, 12, and 18**


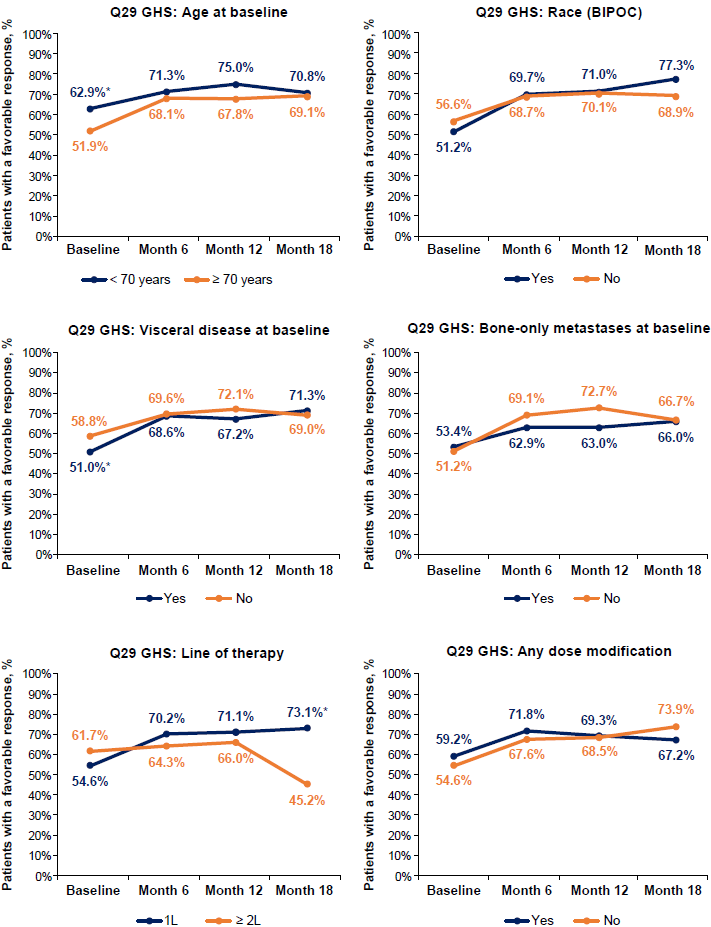


Comparative statistical p values were derived using Fisher’s exact test on the association of EORTC QLQ–C30 domains (favorable/unfavorable) with each subgroup at each specific time point. All Fisher’s exact test subgroup comparisons were not considered to be significant (*P* > 0.05), except baseline age (*P* = 0.003) and visceral disease (*P* = 0.035), and at month 18, line of therapy (*P* = 0.003). * *P* < 0.05 indicates statistical significance.

*1L* first-line, *2L* second-line, *BIPOC* Black, Indigenous, and People of Color (Yes = BIPOC; No = White/not Hispanic or Latino), *EORTC QLQ–C30* European Organisation for Research and Treatment of Cancer Quality of Life Questionnaire Core 30, *GHS* global health status, *Q* question

**Supplementary Fig. S3 Per-label population: Proportions of patients indicating a favorable response on EORTC QLQ–C30 Q30 (QoL) across subgroups at baseline and months 6, 12, and 18**


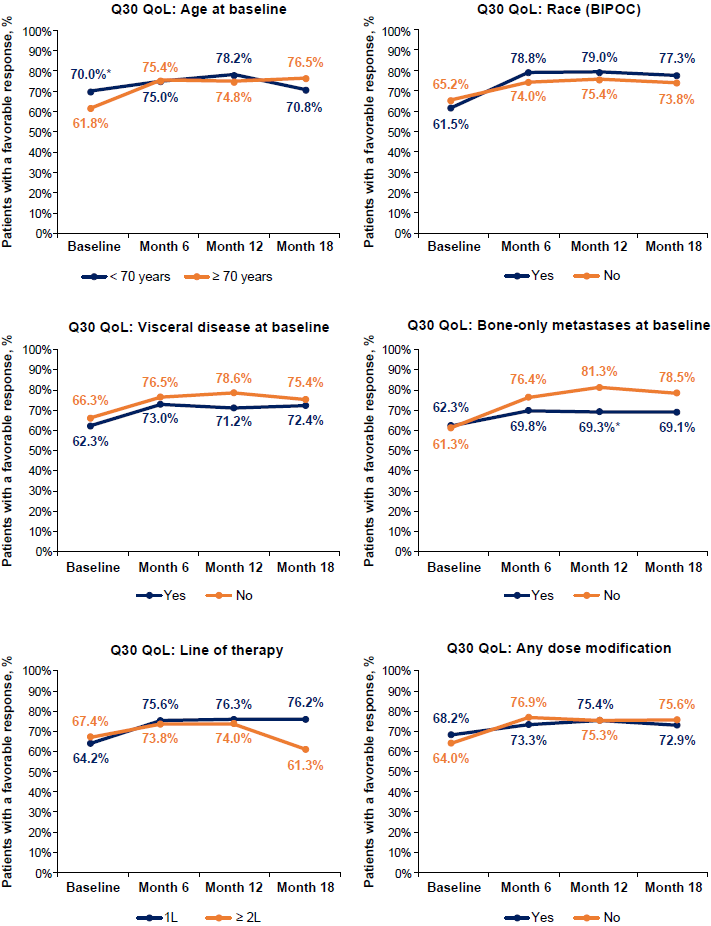


Comparative statistical p values were derived using Fisher’s exact test on the association of EORTC QLQ–C30 domains (favorable/unfavorable) with each subgroup at each specific time point. All Fisher’s exact test subgroup comparisons were not considered to be significant (*P* > 0.05), except baseline age (*P* = 0.021) and at month 12, bone-only metastases (*P* = 0.030). * *P* < 0.05 indicates statistical significance.

*1L* first-line, *2L* second-line, *BIPOC* Black, Indigenous, and People of Color (Yes = BIPOC; No = White/not Hispanic or Latino), *EORTC QLQ–C30* European Organisation for Research and Treatment of Cancer Quality of Life Questionnaire Core 30, *GHS* global health status, *Q* question
